# Gabapentin: Clinical Use and Pharmacokinetics in Dogs, Cats, and Horses

**DOI:** 10.3390/ani13122045

**Published:** 2023-06-20

**Authors:** Federica Di Cesare, Viviana Negro, Giuliano Ravasio, Roberto Villa, Susanna Draghi, Petra Cagnardi

**Affiliations:** Department of Veterinary Medicine and Animal Sciences, Università degli Studi di Milano, Via dell’Università 6, 26900 Lodi, Italy; federica.dicesare@unimi.it (F.D.C.); vivi.negro94@gmail.com (V.N.); giuliano.ravasio@unimi.it (G.R.); roberto.villa@unimi.it (R.V.)

**Keywords:** chronic pain, clinical use, idiopathic epilepsy, neuropathic pain, pharmacokinetics

## Abstract

**Simple Summary:**

Gabapentin is an anticonvulsant drug effective in humans to control neuropathic pain. In veterinary medicine, is extra-label used in combination with other treatments to control seizures when other drugs are not effective, when drugs are toxic, or for neuropathic pain treatment and anxiety. This review aimed to clarify gabapentin use and pharmacokinetic aspects to promote conscious use in dogs, cats, and horses. In dogs, gabapentin was useful in the treatment of epilepsy, as well as chronic, neuropathic, and post-operative pain and anxiety. In cats, it was effective in post-ovariohysterectomy-related pain and in the management of anxiety. In horses, it has been administered as an analgesic for chronic pain. In conclusion, when used in combination with other drugs, gabapentin is an interesting therapeutic option for the treatment of neuropathic diseases and analgesia. Despite its beneficial use in different clinical settings, further trials and pharmacokinetic studies are needed for the definition of an effective dosage regimen through proper pharmacokinetic/pharmacodynamic correlation in dogs, cats, and horses.

**Abstract:**

Gabapentin is an anticonvulsant drug, which presents an established clinical efficacy in human patients for the management of refractory partial seizures, secondarily generalized tonic-clonic seizures, and for the control of chronic neuropathic pain. Gabapentin was synthesized as a structural analogue of the inhibitory neurotransmitter GABA, with GABA-mimetic effects, able to cross the blood–brain barrier. In veterinary medicine, is extra-label used in combination with other treatments to control seizures when other drugs are no longer effective or become toxic or for neuropathic pain treatment and anxiety. This review aimed to clarify gabapentin use and pharmacokinetic aspects to promote conscious use in dogs, cats, and horses. In dogs, gabapentin was beneficial in the treatment of epilepsy, as well as chronic, neuropathic, and post-operative pain, as well as anxiety. In cats, it showed efficacy in post-ovariohysterectomy-related pain and in anxiety management. In horses, gabapentin has been administered as an analgesic for chronic pain management. In conclusion, when used in combination with other drugs, gabapentin can be considered an interesting therapeutic option for the treatment of neuropathic diseases and analgesia in postoperative and chronic pain. However, despite its beneficial use in different clinical settings, further trials and pharmacokinetic studies are needed for the definition of an effective dosage regimen through proper pharmacokinetic/pharmacodynamic correlation in dogs, cats, and horses.

## 1. Introduction

### 1.1. Background

**Figure 1:**
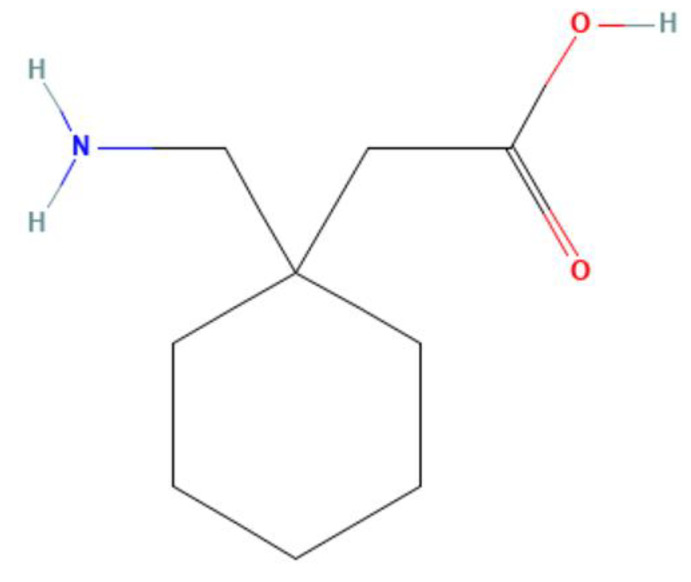
Chemical structure of gabapentin [2].

Gabapentin, 1-(aminomethyl)-cyclohexaneacetic acid (Neurontin^®^ and generic brands), is a water soluble, bitter-tasting, white crystalline substance with a cyclohexane ring incorporated [1] (Figure 1).

It is an anticonvulsant drug synthesized nearly 40 years ago and first approved in 1993 for the treatment of post-herpetic neuralgia in human patients that later proved to be clinically effective in humans suffering from refractory partial seizures and secondarily generalized tonic–clonic seizures. Nowadays, in human medicine, it is approved also for the management of diabetic neuropathy and as adjunctive therapy in the treatment of partial onset seizures, with and without secondary generalization, in adults and pediatric patients with epilepsy. It shows rare adverse effects and few drug interactions with other anticonvulsant agents [3,4,5,6]. After being approved, it was also recognized to be effective as an analgesic in reducing symptoms in human patients with neuropathic pain and was included in the first-line treatment for this medical condition [7]. For its analgesic uses in neuropathic conditions, including neuropathic pain associated with spinal cord injury, complex regional pain syndrome, or others, it is relevant to highlight that all of these are extra-label use and evidence remains extremely limited for neuropathic pain conditions other than post-herpetic neuralgia and diabetic neuropathy [8,9].

Initially, in dogs and cats, gabapentin was used extra-label as an antiepileptic drug, but now it is more commonly used for chronic and neuropathic pain management in companion animals, despite the lack of evidence of efficacy and the lack of correspondence of neuropathic pain conditions in human and animals. However, in dogs, gabapentin showed favourable effects in the treatment of epilepsy [10,11], chronic, neuropathic and post-operative pain, [12,13,14] and anxiety [15,16]. In cats, it showed efficacy in post-ovariohysterectomy-related pain [17,18] and in anxiety management [19,20,21,22]. In horses, gabapentin has been administered as an analgesic for chronic pain management with variable results that suggest the need for further studies. Therefore, the aim of this review is to clarify gabapentin use and pharmacokinetic aspects to promote rational use of the drug in dogs, cats, and horses. 

### 1.2. Review Methodology

The articles included in this review were primarily sourced from PubMed, Medline, Scopus, and Google Scholar databases. To identify and categorize all the significant scientific articles, the authors employed keywords encompassing different categories, such as “Gabapentin”, “Veterinary Medicine”, “Pharmacokinetics”, and “Clinical Effects”, considered as both abbreviations and full names. Additionally, the references cited in the published literature were carefully examined to ensure that they aligned with the aim of the search. The scientific articles dealing with the use of gabapentin in dogs, cats, and horses were included, together with references in which its pharmacokinetic profile was investigated in the same species. From all the retrieved references, articles in which gabapentin had been administered in this species for experimental purposes were excluded, with the exception of experimental pharmacokinetic articles.

## 2. Mechanism of Action

Gabapentin was originally synthesized as a structural analogue of the inhibitory neurotransmitter γ-aminobutyric acid (GABA), with GABA-mimetic effects and able to penetrate the blood–brain barrier. Unlike benzodiazepines or barbiturates, the effective action of gabapentin does not seem to involve central GABAergic functions, either by receptor interaction or by influence with the turnover of the neurotransmitter [5,23]. Despite its chemical structure, gabapentin is neither a GABA—A, nor a GABA—B agonist, nor it is converted metabolically into GABA [5,24,25]. It was later hypothesized that gabapentin selectively activates presynaptic GABA—B heteroreceptors on glutamatergic terminals, but not GABA—B autoreceptors on GABAergic terminals [23]. 

The gabapentin mechanism of action is not completely elucidated, yet, thus contributing to its complexity. It is, hereafter, described based on the different clinical uses reported in companion animals. 

### 2.1. Gabapentin as an Anticonvulsant

Seizures are frequently recurrent in veterinary neurology and are affect up to 5% of the dog population, depending on breed predisposition. Additionally, when dogs and cats are not treated for recurrent seizure activity or are treated with sub-therapeutic doses of anticonvulsant drugs, they tend to experience a worsening of their clinical condition [26]. Gabapentin was not shown to interact with voltage-dependent sodium channels, which are the site of interaction of several antiepileptic drugs, and it did not exhibit affinity for common neurotransmitters receptors (i.e., glutamate or glycine), enzymes, or other ion channels [5,25]. The mechanism of action, as an anticonvulsant agent, has not been fully clarified yet [27,28], and the drug was described as binding neurons in the outer layer of the cortex at distinct sites from other anticonvulsants [29], and it was speculated that gabapentin might exert a role in the modulation of neuronal voltage-gated Ca2+ channels (VGCCs), as shown in pig brain cells [24]. Furthermore, it is now accepted that gabapentin binds with high affinity to α2δ-1 subunits of the VGCCs, and this binding interaction is believed to account for the therapeutic activities of the drug. Additionally, the inhibitory effect of gabapentin on presynaptic calcium channels may induce a reduction in the release of excitatory neurotransmitters, which can be seen as an interesting mechanism to explain the antiseizure activity of this molecule, although the experimental evidence is not fully supportive [30,31]. Recently, it has been proposed that the α2δ-1 subunit associates with N-methyl-D-aspartate receptors, and their inhibition could also contribute to the antiseizure activity of gabapentin [9,30].

### 2.2. Gabapentin as an Analgesic for Neuropathic Chronic Pain

Chronic Neuropathic Pain (CNP) is a complex phenomenon associated with the disease or injury of the peripheral or central nervous system [32,33], and, because of its complexity, it is considered difficult to treat without a multimodal analgesic approach [34,35,36].

In the last two decades, the interest in the treatment of pain, including CNP, has been growing in companion animal therapy, acquiring a central role [37]. Nevertheless, except for some behavioural changes, CNP is still difficultly recognized, mainly due to the limited background of veterinarians in its diagnosis, as well as to the absence of a gold standard for a universal definition of the pain scale [36,37,38]. 

After the assumed successful use in some clinical settings, gabapentin was more deeply investigated and probably due to its highly specific binding to the α2δ-1 auxiliary subunit of the voltage-gated Ca2+ channel, it was suggested to have analgesic properties in the management of variable CNP conditions in dogs [12,13,14,39], cats [40,41,42], and horses [43,44,45]. This specific binding was first described in a study by Gee and colleagues in 1996, where they tested the drug in vitro on pig brain cells [24]. This study tried to explain the anticonvulsant mechanism of action of gabapentin, but it actually ended up defining the binding, whose modulation was later suggested to be fundamental in managing neuropathic pain [3,46]. 

Neuronal VGCCs consist of three subunits: the α1 pore-forming subunit, the intracellular β subunit, and the α2δ subunit, consisting of two disulphide-linked polypeptides encoded by the same gene [46,47]. The gabapentin interaction with this high-affinity binding site may provide a tool for the drug to mediate voltage-gated Ca2+ influx, by indirectly influencing the functional interaction between the accessory α2δ and the pore-forming α1 subunits of Ca2+ channels [3,48]. By indirectly blocking the influx of Ca2+ into the cell, gabapentin causes a modulation of the synaptic transmission and an antiallodynic effect. However, its analgesic effect is expressed in the case of injuries with CNP associated with upregulation of the α2δ-1 subunit, frequently occurring in damaged sensitive nerve fibers. It is proved that nerve injury results in a marked increase in the α2δ subunit before the onset of allodynia [3,46]. 

The Patel et al. study (2000) demonstrated that the antinociceptive action of gabapentin involves a pre-synaptic site of action, and the drug was able to modulate neurotransmission in the spinal cord of hyperalgesic animals suffering from diabetic neuropathy [28]. 

It was later confirmed that gabapentin also has a supraspinal site of action, in which noradrenaline (NA) and GABA brain release are involved [49,50,51,52]. 

**Figure 2:**
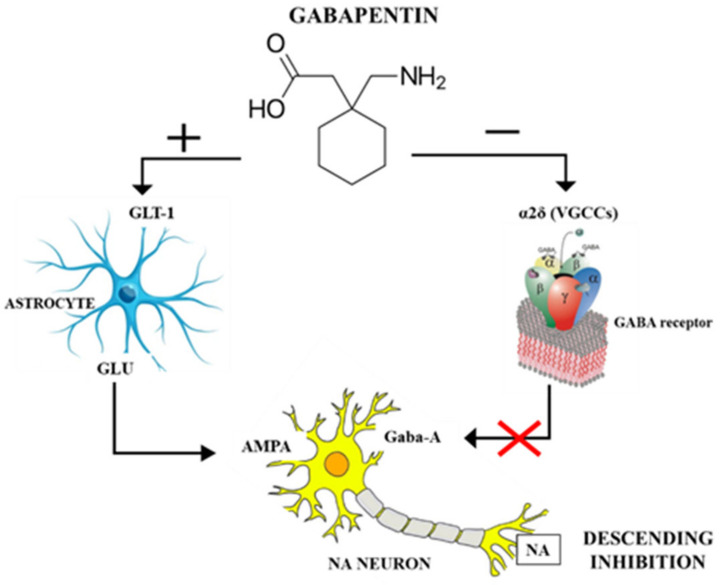
Mechanism of action of gabapentin in the locus coeruleus. GLT-1: glutamate transporter 1. α2δ: subunit of voltage-gated Ca2+ channels (VGCCs). Glu: glutamate. GABA: neurotransmitter gamma-aminobutyric acid. Gaba-A: GABA receptor A. AMPA: (α-amino-3-hydroxy-5-methyl-4-isoxazolepropionic acid) ionotropic transmembrane receptor for glutamate. NA: noradrenalin.

Recently, Hayashida and Obata (2019) [53] proposed a double action: gabapentin would interact in the locus coeruleus (LC) with the α2δ subunit of VGCCs to reduce pre-synaptic GABA release and, at the same time, it would activate glutamate transferase-1 (GLU-1)-dependent mechanisms to induce glutamate (Glu) release from LC astrocytes, thus increasing LC neuronal activity to activate descending inhibition (Figure 2) [53]. Hence, gabapentin acts on LC by blocking VGCCs and increasing extracellular Glu. The latter is able to activate the descending noradrenergic system, which terminates in the lumbar spinal cord, where the NA released interacts with α2-adrenergic receptors to reduce the transmission of nociceptive information. This is actually a common neuronal pathway to inhibit chronic pain, and it is also at the basis of the antidepressant and analgesic mechanisms of action, where gabapentinoids activate the LC, whereas antidepressants inhibit the reuptake of NA in the synaptic cleft, both resulting in increased NA levels in the spinal cord [53]. Gabapentin and other gabapentinoids, on the other hand, did not demonstrate an antinociceptive effect against acute pain in experimental conditions [54,55] and in veterinary clinical settings [56,57,58].

### 2.3. Gabapentin as an Anxiolytic

In animal models, gabapentin was investigated to predict its utility in anxiety treatment [59]. It displayed anxiolytic-like action during the rat conflict test, the mouse light/dark box, and the rat elevated X-maze test. Moreover, it induced behavioural changes, suggestive of anxiolysis in the marmoset human threat test [59]. Widely used in human medicine, gabapentin has shown very beneficial properties in the control of anxiety, but also in the treatment of bipolar disorder, post-traumatic stress, and other psychiatric conditions [60,61,62,63]. Despite the actual anxiolytic mechanism of gabapentin remaining unknown, it was speculated that the experimental results in animal models were achieved due to the binding of the drug to the α2δ subunits of VGCCs, which would result in reducing neurotransmission fear circuits, which are pathologically activated with anxiety disorders [64]. Nevertheless, in human medicine, there is limited evidence for the efficacy of gabapentin in anxiety disorders [65,66].

The anxiolytic effect of gabapentin was evaluated in dogs and cats with positive outcomes in storm phobia, anxiety, and fear reduction, in particular before veterinary visits [15,16,19,20,21,22]. It might be speculated to have an effect on the reduction in the physiological catecholamine release of the “flight and fight” response. As stated above, the gabapentin mechanism of action is not fully understood, and, in particular, the mechanism related to the gabapentin anxiolytic effect requires further specific investigation, and this is also necessary with regards to dogs and cats.

## 3. Clinical Use

### 3.1. Gabapentin in Dogs

The first report on gabapentin use as an anticonvulsant in companion animals was in 2003 at the 16th Annual European College of Veterinary Neurologist Symposium [67]. Since then, gabapentin has been used in dogs in combination with other treatments to control seizures when other drugs are no longer effective or become toxic [10,11]. In fact, there are no studies where gabapentin was used as monotherapy, but only as add-on therapy for patients suffering from partial seizures resistant to conventional therapies.

Consequently, it was tested as adjunctive therapy for refractory idiopathic epilepsy in eleven dogs, with encouraging results concerning the reduction in the number of seizures per week and the number of days with any seizures in a one-week period, although this was obtained in a small population of subjects [11] and as an add-on therapy associated with two of the main anticonvulsants used at that time, phenobarbital (phenobarbitone) and potassium bromide, with a significant increase in the number of patients who showed an extension of the interictal period [10,67].

In 2010, Ghaffari et al. achieved excellent results using gabapentin in association with phenobarbital to control refractory psychomotor seizures in one dog, describing the drug as a safe and effective option for this medical condition, although it was a case study reporting gabapentin use in only one two-year-old male Doberman Pinscher dog [68]. 

In clinical neurology, due to its central origin pain control properties, gabapentin was administered twice to three times a day, in addition to prednisolone, in two Cavalier King Charles spaniel dogs suffering from syringomyelia, showing a reduction in pain severity or ataxia [39]. Despite these encouraging results in the case study of these two dogs, proper randomized, placebo-controlled, masked trials are warranted to assess the analgesic efficacy of gabapentin for the therapy of this medical condition. 

In addition, in a clinical study, describing a method for foramen magnum decompression in dogs with caudal occipital malformation syndrome, gabapentin was described as useful in the control of scratching activity in two dogs in addition or not to glucocorticoids [69]. These data are not exhaustive to claim gabapentin usefulness in scratching activity and can be considered only anecdotal, and thus further studies are warranted.

Gabapentin was also used as an adjunctive treatment to carprofen in clinically affected dogs with Chari-like malformation and syringomyelia, and it was compared to the combination of carprofen and topiramate, showing no significant difference in the quality of life, although less severe side effects were reported for carprofen and gabapentin combination [12]. The limitation of these results might be the lack of a placebo treatment group for obvious ethical reasons [26].

Moreover, Aghighi et al. (2012) showed that gabapentin, in addition to mu-opioid analgesia, was not beneficial in managing pain after thoracolumbar intervertebral disc surgery (i.e., hemilaminectomy), probably due to a dose-related inefficacy [57].

In the management of acute post-operative pain after limb amputation in fifteen dogs, the addition of gabapentin in a multimodal analgesic regimen was not beneficial, although adverse effects were not observed. As stated by the authors, the small sample size and the large use of other analgesics could have hindered the possibility of detecting a beneficial effect of gabapentin [56].

Recently, gabapentin was tested alone or in combination with meloxicam for the management of CNP in a randomized, cross-over, placebo-controlled, partially masked clinical trial, demonstrating its efficacy in reducing the pain burden of dogs affected by different medical conditions, although results might have been biased by the so-called “caregiver placebo effect” [13,58].

Moreover, regarding the management of osteoarthritis-related pain, satisfactory results were recently obtained using gabapentin in a multimodal pharmacological protocol, in which it was combined over a 12-week period with cannabidiol, anti-inflammatory drugs (i.e., firocoxib or prednisolone), and amitriptyline, although the aim of this study was to test the addition of cannabidiol in the multimodal regimen [14]. 

The perioperative administration of gabapentin as an adjuvant for postoperative pain management in dogs undergoing mastectomy did not show differences in the pain scores, but it significantly reduced the incidence of rescue medication [58]. 

After several human ophthalmology studies [70,71,72], gabapentin was first examined in clinically normal dogs to test how it affects intraocular pressure when used in premedication. The results demonstrated that gabapentin can be useful in attenuating the intraocular pressure increase associated with intubation [73]. Conversely, in another study by Shukla et al. (2020) [74], this drug showed a non-clinically meaningful decrease in intraocular pressure and no clinically adverse effects on tear production, pupillary diameter, tear film stability, or corneal sensitivity in healthy dogs [74]. The contrasting results might be related to the fact that dogs were not anaesthetized in the latter study, in which it was also reported that gabapentin did not provide an analgesic effect on the cornea [74]. 

Gabapentin has recently been shown to reduce the Minimum Alveolar Concentration (MAC) of isoflurane when administered orally to dogs as premedication with no effect on hemodynamic variables or vital parameters [75]. Although other factors (e.g., hypothermia, hypercapnia, and hypoxemia) may have contributed to the lower MAC, the reduction in isoflurane requirement is a promising finding, and further studies are warranted to clarify this aspect. 

Moreover, gabapentin produced positive effects on storm phobia when administered orally as a single dose 90 min prior to exposure. This work, while requiring further investigation in a larger population with concomitant behavioural evaluation, would seem to confirm the anxiolytic effect of the drug, as already demonstrated in human patients [15,75]. Another study in dogs reported the effectiveness of a single 50 mg/kg gabapentin oral dose in reducing signs of anxiety, expressed as lip-licking behaviour, related to clinical examination without severe adverse effects [16]. 

Episodes of ataxia and sedation have been occasionally recorded following the administration of gabapentin [10,11,15,76]. Table 1 schematically shows the results obtained from the mentioned papers.

### 3.2. Gabapentin in Cats

Initially, there was only anecdotal information regarding gabapentin use in cats and no information on the safety or efficacy of long-term gabapentin administration. More recently, in feline internal medicine, gabapentin has been investigated mainly for its antinociceptive or anxiolytic effects, but, still, scarce information about gabapentin safety or efficacy as an anticonvulsant agent in cats is available [26].

In two case studies, gabapentin was successfully employed as an adjuvant in the management of hyperalgesia and allodynia in two cats with multiple fractures following the partial failure of opioid and NSAIDs therapy [42] and in one cat for wound debridement in a multimodal analgesia for perioperative pain, since neuropathic pain was suspected [77]. Furthermore, when used as an antinociceptive in long-term treatment for musculoskeletal injuries and head trauma in three cats, the drug showed no side effects and was well tolerated at recommended doses [41]. Obviously, the nature of these reports supports the need to explore this application more thoroughly with appropriate randomized, placebo-controlled, masked trials.

Steagall et al. (2018) [18] reported gabapentin to be potentially beneficial in managing post-operative acute pain in association with opioids, even if the results showed no agreement in pain levels according to the two scales used due to the small sample size evaluated [18]. Improvements in pain levels were detected only with one pain scale, consequently, the authors suggested to better explore gabapentin use for acute post-operative pain management with further studies [18]. 

For the control of chronic osteoarthritis-related pain, gabapentin was associated with improvement in owner-identified impaired activities, but with a lack of improvement in the owners’ reported quality of life, probably due to the high incidence of sedative effects [40,78]. In a preliminary study on transdermal administration of gabapentin in geriatric cats, drug systemic absorption and a decrease in pain scores were observed [78]. Conversely, gabapentin administered at three different doses did not affect the thermal threshold in healthy cats and, therefore, was not able to provide thermal antinociception [79]. Larger studies, cross-over, or including control cats, involving other nociceptive models or using blinded evaluation of the potential analgesic effect, are recommended for a better understanding of gabapentin’s role in the treatment of pain in cats.

Several feline studies have highlighted that stress during transport to the veterinary clinic often makes the animal untreatable and not compliant and thus have tested gabapentin for the management of anxiety disorders. The single oral administration of gabapentin before the visit was deemed effective, also attenuating fear responses without producing sedation [19,20,21,80,81].

Conversely, Hudec et al. (2020) [82], by measuring cortisol and glucose concentrations, tested whether gabapentin, given pre-appointment to healthy cats, altered stress before and during intradermal testing [82]. They observed that gabapentin was not able to reduce cortisol and glucose concentrations, but, as stated by owners and veterinarians, decreased stress-induced responses, and this effect was explained by its sedative effect [82].

Gabapentin was also tested as an anxiolytic in clinically healthy cats to assess the level of sedation, its effects on non-invasive blood pressure and heart rate, and to evaluate echocardiographic measurements of chamber size, as well as systolic and diastolic function. The drug turned out to be a good therapeutic option when orally administered before a cardiology examination, with mild sedative effects and minimal decrease in the echocardiographic measurements of systolic function [22]. Similarly, gabapentin given orally to healthy cats 70 min before echocardiography effectively increased compliance without causing substantial changes in cardiovascular and echocardiographic parameters [83]. 

In 2020, the gabapentin appetite-stimulating effect was evaluated in comparison to mirtazapine in healthy cats during the postoperative period, recording an increase in food intake in the gabapentin group compared to the placebo group. According to this study, gabapentin and mirtazapine produced similar effects on food intake, encouraging their use as appetite promotors [17].

Occasionally as in dogs, ataxia and sedation have also been reported in cats after gabapentin administration [31]. Finally, in contrast to dogs, gabapentin did not have a detectable effect on the MAC of isoflurane in cats [84]. Table 2 summarizes the clinical uses of gabapentin in this species.

### 3.3. Gabapentin in Horses

In horses, conditions associated with neuropathic and chronic pain include laminitis, arthritis, idiopathic headshaking, and navicular syndrome [86,87,88].

In 2007, gabapentin was first used in one horse with a suspected neuropathic injury: a pregnant Belgian mare developed post-anaesthetic neuropathy and signs of intractable pain following colic surgery. After several treatments with flunixin meglumine, butorphanol, xylazine, detomidine, and lidocaine, with no effectiveness, the addition of gabapentin to the therapy was attempted with a positive clinical outcome and clinical signs of pain reduction, at the dosage of 2.5 mg/kg orally every 8 h for 24 h and subsequently decreased to every 12 h for three days and again every 24 h for two days with mild sedation as exclusive side effect [45]. In that study, the authors considered the gabapentin addition beneficial in managing the mares’ pain, despite the dosage employed nowadays being considered to be unlikely able to reach the effective analgesic concentrations reported in humans [89,90]. It is possible that gabapentin might have contributed to decreasing the global pain level experienced by the mare within the multimodal therapeutic approach. The use of gabapentin in this mare was also deemed affordable, on the contrary, at the higher dose suggested by Gold et al. (2022) [90] of 120 mg/kg every 12 h. Its feasibility is questionable, both because of the costs and because of the difficulties in preparing a very high number of tablets for administration [90].

In another case description, gabapentin as adjunctive therapy at 5 mg/kg orally markedly reduced violent head-shaking behaviour in one horse treated for temporohyoid osteoarthropathy within 48 h from its administration [44]. 

For chronic pain, gabapentin was ineffective as a monotherapy for the treatment of chronic lameness [91], and it did not improve subjective or objective measures of lameness in horses with chronic thoracic limb musculoskeletal pain when used alone, but, conversely, it reduced pain when used in combination with firocoxib [43]. Moreover, it did not show adverse effects, i.e., cardiovascular, sedative, and behavioural effects or changes in physiologic or biochemical variables when used alone at the oral dose of 20 mg/kg or at repeated oral doses up to 120 mg/kg in healthy horses [90,92].

Undoubtedly, the use of gabapentin for managing chronic neuropathic pain and related conditions could represent an attractive opportunity. However, further randomized, placebo-controlled, masked trials are necessary to test the efficacy of this drug for managing these conditions. In addition, based on the available information, the use of gabapentin in horses could be contemplated as an adjuvant to traditional drugs in the treatment of chronic and neuropathic pain, within a multimodal analgesia approach that includes cyclooxygenase inhibitors and tramadol [93].

Table 3 shows a summary of studies performed, to date, on gabapentin in horses.

## 4. Pharmacokinetics (PK)

Gabapentin is a human medication, primarily formulated in tablets and capsules to be taken orally. In veterinary medicine, since this drug is not licensed, the prescription is defined as extra-label. Given the commercial existence of only human formulations and the difficulty in finding appropriate extemporary formulations, the use of gabapentin in veterinary medicine may be variably practical when in relation to the size of the species considered and the therapeutic scheme adopted [89,90,94,95,96].

Gabapentin, depending on its physicochemical properties and its ability to be absorbed, distributed, metabolized, and excreted, is to date assigned to Class III of the Biopharmaceutics Drug Disposition Classification System (BDDCS) [97]. As many drugs administered almost exclusively orally, gabapentin presents a variable bioavailability, depending on species, feeding behaviours, and other factors [25,98]. In particular, the presence and saturation capacity of drugs’ intestinal transporters is considered a critical factor for this route of administration [99]. In veterinary medicine, the presence of gabapentin transporters has been suggested to vary in relation to the species, together with the metabolic pathways involved in the biotransformation of the drug [25,98]. With the reported exception of dogs, gabapentin is not metabolized by the liver, and it is normally excreted in its unchanged form in urine [25,100]. This kinetic behaviour could result in specific criticisms for certain categories of subjects (e.g., geriatrics, subjects with chronic hepatic and renal disease). The first studies on gabapentin are dated, but the drug is being progressively included in veterinary long-term therapy. Hence, recently, there has been an increased number of new studies to better explore its PKs.

### 4.1. Pharmacokinetics in Dogs

As reported above, in dogs, a lot of studies are focused on gabapentin effects for the control of epilepsy, chronic, neuropathic or post-operative pain, and anxiety, but, to date, only a few studies have examined its pharmacokinetic properties. In this species, gabapentin is rapidly absorbed, metabolized to N-methyl-gabapentin, and eliminated in its unchanged form in urine [25,100], and the oral bioavailability at a dose of 50 mg/kg was reported to be 80% [25].

In the most recent study, gabapentin was administered orally at a dose of 10 mg/kg on day 1, and at 20 mg/kg on day 2, to six healthy greyhound dogs in order to determine the drug pharmacokinetic profile [96]. For the 10 mg/kg and 20 mg/kg doses, the mean maximum concentrations (Cmax) were 8.54 and 13.22 µg/mL, respectively, attained at mean times to maximum concentration (Tmax) of 1.31 and 1.51 h, and the mean terminal half-lives were 3.25 and 3.41 h. The efficacy of the drug was not evaluated, but the authors considered that efficacy is associated with a plasma concentration of 2 µg/mL in human patients resistant to anticonvulsant therapy, and they thus suggested that 10–20 mg/kg every 8 h would maintain 2 µg/mL plasma concentration in dogs [96]. It has to be highlighted that the effective concentration of gabapentin in animals is unknown. In humans, the half-maximal effective concentration (EC_50_) for treating neuropathic pain is 5.4 μg/mL [101], and a study in rats suggests that the EC_50_ ranges from 1.4 to 16.7 μg/mL for the treatment of inflammatory hyperalgesia [25,102]. No side effects were noted at the above dosages, other than poorly formed stools not directly attributable to the drug intake [96]. The recommended doses in clinical practice are in Table 4 [103]. The main pharmacokinetic parameters in dogs, cats and horses are summarised in Table 5.

### 4.2. Pharmacokinetics in Cats

Nowadays, gabapentin PKs in cats are still poorly investigated, and the literature reported quite different results, even though similar doses were evaluated. The first study by Siao et al. (2010) [94] aimed to determine the PKs of gabapentin in cats receiving 4 mg/kg IV or 10 mg/kg orally [94]. In the study by Adrian et al. (2018) [95], gabapentin was administered by an IV bolus at the dose of 5 mg/kg, orally at 10 mg/kg as a single dose or twice a day for 2 weeks, or as a transdermal gel at 10 mg/kg in serial order [95]. In the study by Adrian et al. (2018) [95], the transdermal formulation was also explored, but the drug concentrations were too low to be modelled with appropriate pharmacokinetic analysis, probably because of the poor absorption of the gabapentin preparation used [95]. Conversely, the results from the most recent study regarding transdermal gabapentin use in cats appeared to be promising and encouraged further investigations on the use of this alternative administration route [78]. Indeed, the results provided by Slovak and Costa (2021) [78], using gabapentin in a 10% or 20% Lipoderm base transdermal preparation, clearly demonstrated that this drug can permeate feline skin in vitro and can be systemically absorbed in vivo, advocating the need to deepen the knowledge on this administration route [78].

Finally, the study by Quimby et al. (2022) [104], after performing a PK study in healthy subjects, used the profile to explore gabapentin serum concentrations in cats affected by chronic kidney disease, suggesting the need for a dose adjustment due to the impaired renal excretion of the drug [104]. To date, there are no data in cats describing the optimum plasma concentration for gabapentin, but, considering the effective concentration in humans or rats [101,102], it has been hypothesized that the effective dose could be approximately 8 mg/kg four times a day [95]. The recommended doses in clinical practice are reported in Table 6 [103]. The main pharmacokinetic parameters are presented in Table 5.

### 4.3. Pharmacokinetics in Horses

Pharmacokinetic studies in the horse reported that orally administered gabapentin is poorly absorbed, with a mean oral bioavailability of about 16%, a much lower value than that reported in the literature for humans (29–83%), rats (79–83%), dogs (80%), and cats (88–94%) [6,25,92,94,95]. It was hypothesized that the low bioavailability in horses could be due to the lower stability or solubility of the oral formulations, as well as the saturation of intestinal transporters or the larger dose that might bypass the absorption window in the small intestine where the transporters are located [92]. In addition, in humans, it is known that the absorption of this drug occurs in the proximal small bowel, where it enters into the bloodstream through the saturable L-amino acid transport system. Hence, due to the competition with L-leucine for these transporters, gabapentin could result in reduced absorption [106,107]. The bioavailability of gabapentin in humans would appear to be proportionally higher at lower doses, with a nonlinear increase between the plasma concentration and the administered dose [106,107,108]. The time to maximum drug concentration in plasma (Tmax 1.41 h) falls within the range of values proposed for other species [94,96,105]. Median plasma terminal half-life reported in horses (3.4 h) by Dirikolu et al. (2008) [105] resulted very similar to that reported by KuKanich and Cohen (2011) [96] in dogs (3–4 h) at the oral dose of 5 mg/kg, whereas Terry et al. (2010) [92] indicated a mean value of 7.7 h following an oral administration at 20 mg/kg [92,96,105]. Results obtained after intra-gastric administration with increasing doses (from 10 to 160 mg/kg) showed a different plasma terminal half-life, ranging from 2 to 15.7 h. Moreover, gabapentin plasma concentrations increased across dose levels with no proportionality with dose escalation for the area under the concentration-time curve from zero to infinity and maximal plasma concentration. These values may further support the theory of intestinal transport saturation [89]. The efficacy of repeated dose administration higher than 20 mg/kg has yet to be confirmed, but this dosage is not reported to be associated with any cardiovascular or behavioural effects that would cause its preclusion as a future therapy [89,92]. Considering the gabapentin effective plasma concentration in the rat for the treatment of inflammatory hyperalgesia (16.7 µg/mL) [100,102], a therapeutic dosage in the horse that maintains the same plasma concentration levels could be 10 mg/kg every 8 h, 20 mg/kg every 12 h, or 80 mg/kg every 24 h [89]. Recently, another study by the same authors [90] reported a dosage regimen of 120 mg/kg per 12 h, orally, to reach the effective analgesic concentration reported in humans. This study also explored the occurrence of clinical and biochemical side effects, suggesting that the evaluated scheme would be safe for horses, since changes in physiologic or biochemical variables were not observed, such as signs of ataxia or sedation, which are the reported adverse effects of gabapentin in dogs and cats [31,90]. Nevertheless, the limitations of the studies are the small sample size (six healthy horses), the use of human tablets ground and mixed with corn oil and applesauce or molasses that could have limited the drug bioavailability, and the correlation of PK results with efficacy concentration not tested in the horse. Moreover, as already stated, the clinical practicality of such a high dosage regimen is questionable, both for the costs and for the lack of appropriate formulations.

Despite the recent information, further studies are needed to assess, more precisely, the PKs of gabapentin in horses, especially for the definition of an effective dose through proper pharmacokinetic/pharmacodynamic studies, considering the low bioavailability of the drug and to avoid toxic effects. Table 7 reports the recommended doses in equine patients for clinical practice [103]. The information on the likely effective dose of gabapentin stated by Gold et al. (2020) [89] and Gold et al. (2022) [90] is not included in the table of the recommended doses in equine patients, which has since been published [89,90]. The main pharmacokinetic parameters in companion animals are summarised in Table 5.

## 5. Conclusions

Gabapentin has been recently exploited in companion animal medicine to manage different medical conditions. In dogs, it was reputed beneficial in the treatment of epilepsy, chronic, neuropathic, and post-operative pain, as well as anxiety, but, to date, the information needs to be further investigated. In cats, it showed promising outcomes in the management of post-operative pain and also in the treatment of anxiety. In equine internal medicine, it is used as an analgesic for chronic pain with limited sound scientific evidence. Gabapentin’s pharmacokinetic profile has not been extensively studied in companion animals, and further studies combining PKs with efficacy evaluation are strongly encouraged to propose a rational dosing in the different animal species. Despite its proposed beneficial use in different clinical settings, further proper randomized, placebo-controlled, masked trials are warranted to assess the efficacy of gabapentin in dogs, cats, and horses for proposing its thoughtful inclusion in veterinary drugs’ armamentarium.

## Figures and Tables

**Table 1:** Summary of the different studies on oral gabapentin use in canine patients.

Medical Condition	Therapeutic Regimen	Clinical Endpoint	Outcome	Study Type	Reference
Refractory idiopathic epilepsy	An amount of 35–50 mg/kg every 24 h, divided in three times for four months as adjunctive therapy	To assess a change in seizure activity	Beneficial in increasing the interictal period	Prospective clinical trial (n = 17)	[10]
Refractory idiopathic epilepsy	An amount of 10 mg/kg every 8 h for a minimum three months as adjunctive therapy	To assess a change in seizure activity	Beneficial in reducing seizures episodes	Prospective clinical trial (n = 11)	[11]
Refractory psychomotor seizures	An amount of 10 mg/kg every 8 h	To assess a change in seizure activity	Beneficial in resetting seizures episodes	Case report (n = 1)	[68]
Postoperative pain after intervertebral disc surgery	An amount of 10 mg/kg every 12 h, starting from 2 h before anaesthesia to five days after surgery, in addition to opioid analgesia	To assess the effect of adjunctive gabapentin after surgery	Nonbeneficial	Randomized, placebo-controlled, masked trial (n = 63)	[57]
Neurogenic pain from syringomyelia	An amount of 10 mg/kg every 8–12 h, long-term therapy depending on clinical response	Medical treatment for neurogenic pain	Beneficial	Case report (n = 2)	[39]
Pain from Chiari-like malformation and syringomyelia	An amount of 11.5 ± 2.5 mg/kg every 8 h for six weeks with carprofen	Medical treatment for neurogenic pain	Beneficial	Cross-over, masked trial (n = 40)	[12]
Chronic neuropathic pain	An amount of 10 mg/kg every 8 h for seven days alone or in combination with meloxicam every 24 h	Medical treatment for neurogenic pain	Beneficial	Randomized, cross-over, placebo-controlled, partially masked trial (n = 31)	[13]
Postoperative pain after amputation of a forelimb	An amount of 5–10 mg/kg every 12 h, from one day before surgery to three days after in a multimodal analgesic regimen	To assess postoperative pain reduction	Nonbeneficial	Randomized, placebo-controlled, masked trial (n = 30)	[56]
Postoperative pain after mastectomy	An amount of 10 mg/kg prior to surgery and every 12 h for three days after surgery, as adjunctive therapy	To assess postoperative pain reduction	Nonbeneficial in reducing pain, beneficial in reducing the requirement of rescue analgesia after surgery	Randomized, placebo-controlled, masked trial (n = 20)	[58]
Osteoarthritis	An amount of 10 mg/kg every 12 h (first week), 5 mg/kg every 12 h (rest of the period), 12 weeks in a multimodal analgesic regimen	Medical treatment for neurogenic pain and quality of life improvement	Beneficial in association with anti-inflammatory drugs, cannabidiol, and amitriptyline	Prospective, randomized clinical trial (n = 21)	[14]
Healthy dogs	An amount of 50 mg/kg 2 h before anaesthesia in single administration	To assess intraoperative ocular pressure changes	Beneficial in attenuating intubation-related intraocular pressure increase	Randomized, placebo-controlled, masked trial (n = 20)	[73]
Healthy dogs	An amount of 10 mg/kg every 8 h for three days	To assess intraocular pressure, tears production and film stability, pupillary diameter, and corneal sensitivity variation	Nonbeneficial	Randomized, cross-over, masked trial (n = 9)	[74]
Healthy dogs	An amount of 20 mg/kg 2 h before anaesthesia and after seven days	Evaluation of gabapentin effect in premedication on the MAC of isoflurane	Beneficial in reducing the MAC	Randomized, cross-over, masked trial (n = 6)	[75]
Storm phobia and anxiety	An amount of 25–30 mg/kg 90 min before the exposure, single dose	Reduction in fear responses	Beneficial	Cross-over, placebo-controlled, double-blinded trial (n = 18)	[15]
Anxiety and fear	An amount of 50 mg/kg, single dose 2 h before the visit	To create fear-free veterinary visits	Beneficial	Randomized, cross-over, placebo-controlled, double-blinded trial (n = 22)	[16]

**Table 2:** Summary of the different studies on oral gabapentin use in feline patients.

Medical Condition	Therapeutic Regimen	Clinical Endpoint	Outcome	Study Type	Reference
Major trauma from road traffic accident	An amount of 10 mg/kg every 8 h for two weeks in a multimodal analgesic regimen	Managing chronic neuropathic pain	Beneficial	Case report (n = 2)	[42]
Musculoskeletal injuries and trauma	An amount of 6.5 mg/kg every 12 h up to 12 months initially as adjunctive therapy and then as single analgesic at home	Managing chronic neuropathic pain	Beneficial	Case report (n = 3)	[41]
Postoperative pain after ovariohysterectomy	An amount of 50 mg, administered 12 h and 1 h before surgery in combination with buprenorphine	Managing postoperative pain	Beneficial	Randomized, placebo-controlled, masked trial (n = 52)	[18]
Osteoarthritis	An amount of 10 mg/kg every 12 h for two weeks	Managing chronic pain	Beneficial, sedation as unique side effect	Randomized, cross-over, placebo-controlled, masked trial (n = 20)	[40]
Osteoarthritis and dental disease	An amount of 10 mg/kg every 8 h for five days (transdermal route)	Managing chronic pain	Beneficial	Prospective clinical trial (n = 15)	[78]
Discospondylitis-related chronic neuropathic pain	Not reported	Managing chronic neuropathic pain	Not reported	Retrospective clinical trial (n = 17)	[85]
Thermal antinociceptive threshold test	Amounts of 5, 10, and 30 mg/kg, single administration	Determination of the thermal antinociceptive effect of various single doses	Non beneficial	Randomized, cross-over, placebo-controlled, masked trial (n = 6)	[79]
Anxiety and fear	An amount of 9.2–47.6 mg/kg, single administration	To create a fear-free veterinary visits	Beneficial	Randomized, placebo-controlled, double-blinded trial (n = 53)	[19]
Anxiety and fear	An amount of 13.0–29.4 mg/kg, single administration 90 min prior to placing the cat into the carrier	To create a fear-free veterinary visits	Beneficial	Randomized, cross-over, placebo-controlled, masked trial (n = 20)	[20]
Anxiety and fear	An amount of 100 mg/cat ≤ 4 kg, 150 mg/cat ≥ 4.1 kg, single administration 30 min before the visit	To create a fear-free veterinary visits and provoke mild sedation to execute an hemodynamical and cardiological evaluation	Beneficial	Randomized, cross-over, placebo-controlled, double-blinded trial (n = 10)	[22]
Anxiety and fear	An amount of 20 mg/kg, single administration 1 h prior to leaving home	To evaluate the efficacy of gabapentin as an anxiolytic in hyperthyroid patients	Beneficial	Randomized, placebo-controlled, double-blinded trial (n = 10)	[21]
Healthy cats undergoing ovariectomy	An amount of 5 mg/kg, two administrations after surgery, during an 8 h observation period	Evaluation of the appetite-stimulating effect	Beneficial	Randomized, placebo-controlled, double-blinded trial (n = 60)	[17]

**Table 3:** Summary of the different studies on oral gabapentin use in horses.

Medical Condition	Therapeutic Regimen	Clinical Endpoint	Outcome	Study Type	Reference
Neuropathic pain following colic surgery	An amount of 2.5 mg/kg every 8 h for six days	Managing chronic neuropathic pain	Beneficial	Case report (n = 1)	[45]
Healthy	An amount of 20 mg/kg, single dose	To assess cardiovascular, sedative and behavioral effects	Nonbeneficial	Randomized, cross-over trial (n = 6)	[92]
Temporohyoid osteoarthropathy	An amount of 5 mg/kg every 12 h for 10 weeks as adjunctive therapy	Reducing the violent head shaking	Beneficial	Case report (n = 1)	[44]
Chronic lameness	An amount of 5–10 mg/kg every 8 h for 14 days	Managing chronic pain	Nonbeneficial	Randomized, cross-over, placebo-controlled, double-blinded trial (n = 6)	[91]
Chronic lameness	An amount of 20 mg/kg every 12 h for 6.5 days	Managing chronic pain	Potentially beneficial if associated with firocoxib (57 mg/day)	Randomized, cross-over, placebo-controlled, masked trial (n = 14)	[43]

**Table 4:** Recommended doses for dogs in clinical practice [103].

Anticonvulsant Dosage	Neuropathic Pain	Treatment of Behaviour Disorders
10–20 mg/kg every 8 h orally (PO). The higher dose may be needed in some dogs to control seizures.	Start with 5–15 mg/kg every 12 h PO and increase dose gradually to as high as 40 mg/kg every 8–12 h PO if necessary.	5–30 mg/kg up to three times daily. Start at the low end and titrate up to achieve the desired event, but avoid adverse effects. Dose changes should occur about seven days apart. For short-term treatment to achieve anxiolysis, doses have been as high as 30–60 mg/kg 1–2 h before an event that is anticipated to trigger anxiety in a dog.

**Table 5:** Main pharmacokinetic parameters of gabapentin in dogs, cats, and horses expressed as mean (±SD) or median (range min–max).

Dose and Formulation	Bioavaila-Bility (%)	C_MAX_ (µg/mL)	T_MAX_ (h)	Terminal Half-Life (h)	AUC (µg × h/mL)	CL (ml/min/kg)	VD (L/kg)	K_EL_ (1/h)	Effective Dose (Supposed or Proven)	N. of Subjects	Reference
DOG											
10 mg/kg orally (po)-tablets		9.68 (5.32–10.90)	1.50 (0.75–2.00)	3.35 (2.63–3.68)	49.5 (39.77–63.02)	3.49 (2.97–4.26)	0.94 (0.85–1.26)	-	10–20 mg/kg every 8 h	6	[96]
20 mg/kg po-tablets		12.95 (10.70–18.20)	1.75 (1.00–2.00)	3.39 (3.07–3.91)	83.01 (60–151.24)	3.97 (2.65–5.56)	1.20 (0.83–1.48)	-			
50 mg/kg po	80	56.3	1.1	2.2	242	-	-	-		1	[25]
50 mg/kg iv	-	-	-	2.9	367	2.27	0.16	-			
CAT											
4 mg/kg iv-bolus		47.39 ± 5.10 (30.21–62.38)	-	-	-	3.02 ± 0.18 (2.45–3.46)	0.65 ± 0.01 (0.60–0.70)	0.035 ± 0.004 (0.026–0.049)		6	[94]
10 mg/kg po-capsules	88.7 ± 11.1 (49.6–118.3)	7.98 ± 1.05 (4.64–10.55)	1.66 ± 0.3 (1.0–2.92)	2.95 ± 0.3	-	2.99 ± 0.22 (2.45–3.46)	-	0.004 ± 0.000 (0.003–0.003)			
5 mg/kg iv-bolus	-	-	-	3.78 (3.12–4-47)	32.01 (24.61–42.38)	2.67 (1.99–3.31)	0.804 (0.643–1.05)	1.21 (0.87–1.67)		8	[95]
10 mg/kg po-capsules-single dose	94.8 (82.5–122.8)	12.42 (8.31–18.35)	1.05 (0.74–2.11)	3.53 (2.96–4.78)	73.68 (55.71–107.19)	-	-	0.20 (0.14–0.23)	8 mg/kg every 6 h		
10 mg/kg po-capsules-repeated doses	-	14.78 (9.70–18.41)	0.77 (0.58–1.64)	3.90 (3.12–4.51)	77.25 (66.26–121.06)	-	-	0.18 (0.15–0.22)			
20 mg/kg po-capsules single dose	-	24.2 (18.4–27.1)	1.5 (1–2)	4.1 ± 0.5	158 (144–172)	-	-	0.17 (0.14–0.2)		5	[104]
HORSE											
5 mg/kg po	-	0.27 ± 0.02	1.41 ± 0.1	3.40 ± 0.48	1.78 ± 0.26	0.05± 0.01	-	-		4	[105]
20 mg/kg iv		73.0 (66.2–76.3)	-	8.53 (7.06–13.3)	216.6 (195.1–267.5)	1.61 (1.28–1.68)	0.81 (0.66–0.90)	-		6	[92]
20 mg/kg po	16.2 ± 2.8	3.75 (1.89–5.76)	1.00 (0.75–2.00)	7.73 (6.70–11.93)	38.8 (22.9–61.6)	1.45 (1.21–1.6)	-	0.09 (0.06–0.1)			
10 mg/kg nasogastric po-water dissolved tablets	-	1.6 (1.5–2.4)	2.6 (2.0–3.3)	5.5 (3.9–11.3)	31 (27–35)	-	-	0.6 (0.478–0.760)	10 mg/kg every 8 h or 20 mg/kg every 12 h or 80 mg/kg every 24 h	3	[89]
20 mg/kg nasogastric po-water dissolved tablets	-	3.2 (3.2–3.5)	2.7 (2.5–2.9)	7.6 (6.1–8.2)	49 (48–61)	-	-	0.9 (0.467–1.09)		3	
60 mg/kg nasogastric po-water dissolved tablets	-	4.0 (2.8–8.4)	1.9 (1.5–2.4)	7.2 (2.1–13.8)	79 (52–150)	-	-	1.5 (0.747–2.07)		6	
80 mg/kg nasogastric po-water dissolved tablets	-	6.7 (3.2–10.0)	2.2 (1.0–2.7)	6.0 (4.1–11.9)	110 (69–180)	-	-	0.8 (0.506–2.818)		6	
120 mg/kg nasogastric po-water dissolved tablets	-	8.4 (4.8–15)	1.8 (1.4–3.7)	7.9 (13.3–15.7)	150 (120–220)	-	-	0.8 (0.792–1.81)		6	
160 mg/kg nasogastric po-water dissolved tablets	-	8.5 (5.0–11.0)	1.4 (1.0–2.6)	11.5 (4.1–13.9)	160 (110–230)	-	-	2.3 (0.522–3.492)		6	
40 mg/kg po-tablets mixed with corn oil and applesauce or molasses every 12 h for 14 days	-	7.6 (6.2–11) first dose 8 (6.6–11) last dose	1 (1–2) first dose 0.4 (0–8) last dose	11 (6–30) first dose 15 (13–29) last dose	146 (104–400) after last dose	-	-	-	120 mg/kg every 12 h	6	[90]
120 mg/kg po-tablets mixed with corn oil and applesauce or molasses every 12 h for 14 days	-	9.9 (6.1–27) first dose 22 (14–33) last dose	1 (1–2) first dose 0.0 (0.0–0.3) last dose	7.2 (6.2–19) first dose 20 (12–22) last dose	550 (270–650 after last dose)	-	-	-			

**Table 6:** Recommended doses for cats in clinical practice [103].

Anticonvulsant Dosage	Neuropathic Pain	Treatment of Anxiety
An amount of 5–10 mg/kg every 12 h orally (PO) The dosage has been increased to 20 mg/kg every 8–12 h in some cats to control seizures.	An amount of 5–10 mg/kg every 12 h PO	An amount of 100 mg per cat PO (20–30 mg/kg) Administer 90 min prior to stressful events (such as a veterinary visit) with a peak effect at 2–3 h.

**Table 7:** Recommended doses for equine patients in clinical practice [103].

Neuropathic Pain	Laminitis
2.5 mg/kg every 12 h orally (PO), increased to higher doses if needed (see laminitis dose).	An amount of 2.5 mg/kg every 8 or 12 or 24 h, increased to higher doses when needed. Doses of 10 mg/kg and higher administered every 8–12 h reached concentrations that are effective in other animals.

## Data Availability

As this is a review article, no new data are presented.
